# STEM ability perceptions, basic needs satisfaction, and intrinsic motivation in adolescents: The role of inclusive perceptions in self-determination

**DOI:** 10.1371/journal.pone.0318266

**Published:** 2025-03-11

**Authors:** Angelina Joy, Adam Hartstone-Rose, Jerica Knox, Channing J. Mathews, Jacqueline Cerda-Smith, Kelly Lynn Mulvey

**Affiliations:** 1 Department of Psychology, North Carolina State University, Raleigh, North Carolina, United States of America; 2 Department of Human Development and Family Science, Purdue University, West Lafayette, Indiana, United States of America; 3 Department of Biological Sciences, North Carolina State University, Raleigh, North Carolina, United States of America; 4 Department of Psychiatry, University of Maryland, Baltimore, Maryland, United States of America; 5 Department of Psychology, The University of Virginia, Charlottesville, Virginia, United States of America; University of Tartu, ESTONIA

## Abstract

Current work suggests that basic psychological needs are related to higher intrinsic motivation, which in turn, can promote more positive academic outcomes. However, few studies have examined how perceptions around one’s abilities in science, engineering, technology, and math (STEM) are related to intrinsic motivation and what role needs satisfaction plays in this association. This study assessed adolescents’ (*N* = 285, 56.1% female, *M*_age_ = 15.76 years, *SD* = 1.24) STEM ability perceptions, basic needs satisfaction, and intrinsic motivation. A path analysis was used to examine the association between STEM ability perceptions, basic needs satisfaction, and intrinsic motivation in adolescents. Inclusive perceptions of the STEM abilities of historically underrepresented groups (i.e., girls and minoritized ethnicities) were positively associated with basic needs satisfaction and basic needs satisfaction was positively associated with intrinsic motivation. There was also a positive indirect effect from inclusive perceptions of STEM abilities to intrinsic motivation through basic needs satisfaction. These findings suggest that schools should focus on promoting inclusive perceptions in order to bolster adolescents’ basic needs satisfaction, which could have carry-on effects on intrinsic motivation.

## Introduction

Over the last decade, groups historically excluded from science, technology, engineering and math (STEM), including women and people from traditionally underrepresented and minoritized ethnicities (URM), have earned more STEM degrees, but remain relatively underrepresented in these fields [1]. Although women earned about half of all bachelor’s degrees in STEM in the US in 2018, only 22% of students earning engineering degrees and 20% of students earning computer science degrees were women [1]. Additionally, White students earned about 58% of bachelor’s degrees in STEM, whereas URM students earned only 24% of bachelor’s degrees in STEM [1]. These disparities are evident not only in the US but also globally. For example, UNESCO reports that only 35% of STEM students in higher education worldwide are women [2].

**Fig 1:**
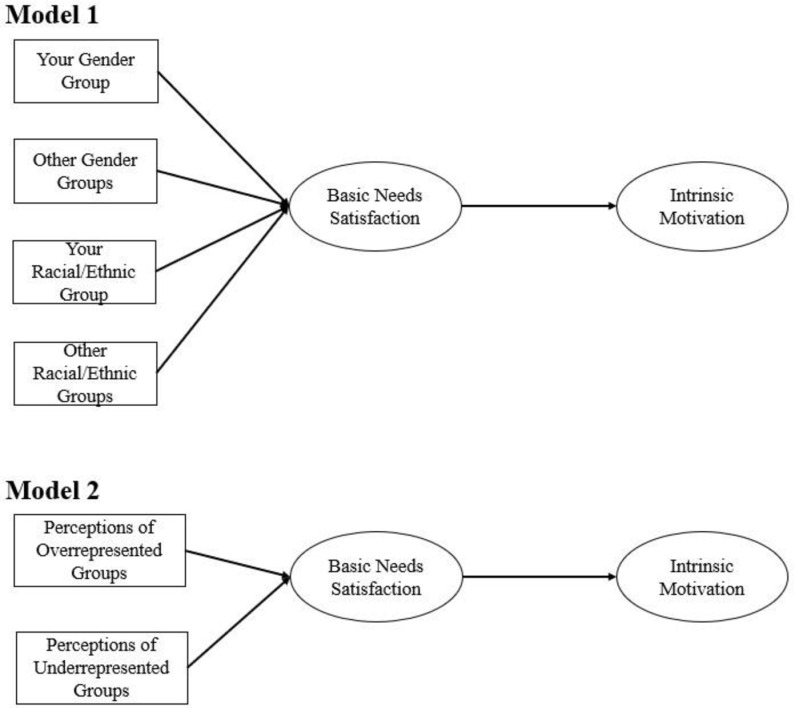
Conceptual models.

Disparities in STEM engagement do not begin in higher education but are evident as early as high school. Although in advanced high school math and science course enrollment there are no *gender* differences, only 9% of Black and 15% of Latinx students enroll in these courses—significantly lower percentages than their White (22%) and Asian (50%) peers [3]. Despite the gender and racial/ethnic disparities evident in college and high school STEM engagement, young children generally start school with positive views of science [4]; yet their interests and feelings of competency in these subjects tend to decline with age, especially for girls and URM individuals [5,6]. This decline could be because STEM-related stereotypes depict girls and those from URM groups as less skilled in these areas [7,8]. With a growing need for trained workers to enter the STEM workforce [9], it is important to determine the factors that promote and limit the pursuit of math and science (the core STEM courses in middle and high school) among adolescents – the age group in which intrinsic motivation for math and science most precipitously declines [10,11]. Furthermore, the fulfillment of student’s basic psychological needs [autonomy, competence, and relatedness; [12,13] has been associated with intrinsic motivation. Thus, research is needed to identify factors that drive or limit students’ perceptions of their basic psychological needs, such as perceptions of who can and should be good at STEM. These perceptions, often rooted in stereotypes about STEM, may be especially important for shaping basic psychological needs as these perceptions can send damaging and exclusionary messages about pursuing STEM interests. Therefore, the aim of this study is to examine the associations between adolescents’ satisfaction of basic needs in their STEM courses, intrinsic motivation in their STEM classes, and perceptions of STEM abilities (see Fig 1).

Conceptual models for self-affiliated STEM ability perceptions (Model 1) and inclusive STEM ability perceptions (Model 2).

### Basic psychological needs

According to self-determination theory (SDT), three basic psychological needs must be met to motivate individuals: autonomy, competence, and relatedness [13]. People must feel in control of their actions and choices (autonomy), capable of learning new skills (competence), and a sense of belonging [relatedness; 13]. This theory explains students’ persistence (motivation) or indifference to science and math courses [14,15], and predicts that if students’ psychological needs are satisfied, they will be motivated to pursue STEM. For example, adolescent Canadian male and female students between 15 and 19 years-old, who reported higher self-efficacy and autonomy-support also reported higher achievement, which led to greater persistence in their math and science courses [15]. Brandenberger, Hagenauer, and Hascher [14] found similar results for adolescents in Switzerland (about 13 years old) who participated in an intervention program based on concepts of SDT. Students who received the intervention reported higher intrinsic motivation in math, compared to the control group [14]. Additionally, basic psychological needs can act as mediators, Plante and colleagues [16] found that the associations between adolescent girls’ gender ability perceptions about math and their achievement outcomes (math grades and career intentions) was mediated by their competence beliefs. Taken together, these findings indicate an association between students’ basic needs satisfaction and many positive STEM outcomes, like intrinsic motivation, that can help support students’ persistence in STEM [14,15] and suggest that perceptions of STEM may play an important role in these relationships.

### STEM intrinsic motivation

Intrinsic motivation for school, characterized by one’s willingness to learn, has been associated with students’ basic needs satisfaction and many academic benefits such as STEM persistence [17,18]. Individuals with high intrinsic motivation are driven by curiosity and interest to acquire new skills or knowledge [12]. High intrinsic motivation in academics can lead to/come with many advantages, such as higher academic achievement, lower academic anxiety, and higher academic performance over time [12,19,20]. Froiland and Davison [21] conducted a longitudinal study of US adolescents (ages 14-17) and found that motivation in math predicted their math achievement and highest-level course taken. Although similar beneficial effects of intrinsic motivation on engagement and achievement have been observed in Black and Hispanic/Latinx populations [22], there have been mixed findings on gender and racial/ethnic differences in intrinsic motivation with some studies reporting no ethnic or gender differences [23] and others reporting that girls and ethnically minoritized students have more intrinsic motivation than do boys and White students [24]. Thus, more research is needed to examine gender and racial/ethnic differences in intrinsic motivation in STEM.

Although intrinsic motivation benefits students of all ethnicities and genders, levels of intrinsic motivation are not consistent and are likely to decline as students get older [10,25]. Otis, Grouzet and Pelletier (25) observed a gradual decrease in intrinsic motivation for Canadian students between 8^th^ grade (aged 13-14) and 10^th^ grade (aged 15-16). These declines are also prevalent for motivation in math and science [10,11]. Gottfried and colleagues [10] discovered a decline in intrinsic motivation for reading, science, and math between the ages of 9 and 17, with math having the greatest decrease. These trends may be explained by the extrinsic school environments that many students experience [26]. Students may feel more pressure to focus on grades and grade point average as they age. This may be particularly salient for students in their last years of high school as they begin to apply for college or consider entering the workforce.

Even though intrinsic motivation has been shown generally to decline throughout school, some factors can help students feel motivated. For instance, Gnambs and Hanfstingl [27] found trends of academic intrinsic motivation gradually declining from the ages of 11 to 16; however, when students’ psychological needs (competence, autonomy, and relatedness) were met, their intrinsic motivation stayed more stable, suggesting that psychological needs may act as a buffer against declining intrinsic motivation. Additionally, school belonging (relatedness) for Dominican children (ages 6 to 11) in New York City directly contributed to their intrinsic motivation despite students’ awareness of negative perceptions of their ethnic group [24]. Based on the SDT [13] and previous recommendations for using SDT to promote more inclusion within STEM [28], creating inclusive environments where students, especially URM and female students, can feel autonomous, competent, and welcomed may help motivate them to engage in school and their STEM classes.

### Perceptions of STEM abilities

One way to foster such inclusive environments is to shape spaces where students perceive that everyone, including their own gender and ethnic groups, can succeed in STEM. Research has highlighted a myriad of factors that impact women and ethnically minoritized individuals in STEM, with one of the most impactful being the prevalence of stereotypes or negative STEM ability perceptions [29]. Some common ability perceptions that individuals may encounter are that Black people are less competent than are White people in academic domains [7,8] or that men are more intelligent than are women [30–32]. Endorsing these ability perceptions, has been negatively associated with youth’s math and science identity and such endorsement increases during adolescence [33].

On the other hand, resisting STEM stereotypes or having more inclusive perceptions, for instance, by believing that groups can excel in STEM, may promote basic needs satisfaction. For example, Plante and colleagues [16] found that adolescent girls’ gender stereotypes about math shaped their competence beliefs (a basic psychological need), which was then related to their achievement outcomes (math grades and career intentions). However, adolescents are exposed to messages about the ability for both their own ethnic/racial and gender groups as well as messages about other groups [7,8]. It may be that both inclusive thinking about STEM abilities of historically underrepresented groups (girls, URM), as well as inclusive thinking about STEM abilities of peers who share your race and gender (perceptions of your gender or racial/ethnic group) help to promote basic psychological needs.

When children are young, they tend to show ingroup favoritism, believing that people from their own gender or racial/ethnic group have higher abilities in academic settings [34]. However, research has shown that endorsement of more stereotypical perceptions (e.g., girls have lower abilities than boys in STEM) throughout adolescence [33] may inhibit students’ future STEM pursuits. Believing that their social groups have lower abilities within STEM may influence how they perceive their own abilities within STEM. Furthermore, marginalized individuals can experience stereotype threat when they feel at risk of conforming to stereotypical perceptions about their social group, which can lead to underperformance on tasks or tests [35–37]. Stereotype threats are common within STEM, especially math domains [36,38,39]. When negative perceptions that depict girls and URM as being less competent are made salient, students from these groups tend to underperform on tests [36,39–41 ]. Therefore, simply being aware of the negative perceptions or understanding that others might endorse such perceptions can lead to underperformance [36]. Additionally, awareness of stereotypes has been related to other outcomes like lower intrinsic motivation and a lower sense of belonging in STEM [24,42]. However, research has yet to examine how thinking inclusively about abilities is related to basic needs satisfaction or what type of inclusive thinking is more important: is having high self-affiliated STEM ability perceptions (thinking that your ethnic and gender group will perform well) more impactful than inclusive STEM ability perceptions (thinking historically underrepresented groups will perform well) on basic needs satisfaction?

### STEM perceptions and self-determination

Some research has shown that endorsing stereotypical perceptions is not directly predictive of intrinsic motivation [43], suggesting there may be factors that could mediate this relationship. One factor may be basic needs satisfaction, which is positively associated with intrinsic motivation [13,14]. Some evidence suggests this may be the case: Grounded in SDT, Nehmeh and Kelly [44] conducted interviews focused on competence, autonomy and relatedness with undergraduate physics students and found that awareness of negative perceptions hindered students’ self-determination in physics. Therefore, research has shown an association between basic needs satisfaction and intrinsic motivation, and between basic needs satisfaction and perceptions however, we have yet to determine if basic needs satisfaction can mediate the association between STEM perceptions and intrinsic motivation. Current research is needed to both re-examine the relationship between STEM perceptions and intrinsic motivation and to identify if STEM perceptions have an indirect effect on motivation through other factors like basic psychological needs.

Research has also suggested that early adolescence may be a time in which youth begin to become more aware of traditional academic perceptions [45] and students underrepresented in STEM, such as ethnic minoritized youth, may be more aware of such perceptions compared to their White counterparts [46,47]. However, adolescence is also a time in which having more inclusive perceptions also becomes more common [48,49], and is related to positive social and academic outcomes as well as psychological well-being [50,51]. Studies have found that girls who resisted traditional math and science gender ability perceptions had higher intentions to pursue math and science compared to girls who did not resist these perceptions [52] and resistance to negative perceptions has also been related to greater persistence on math tasks for women [53]. However, research has not yet examined how inclusive STEM ability perceptions impacts basic needs satisfaction or intrinsic motivation. Research has shown endorsing traditional ability perceptions can have negative impacts for students [36,42,44], but it is unknown if having inclusive ability perceptions can have positive impacts on students’ autonomy, competence, and relatedness within STEM. Moreover, little is known about which types of perceptions might matter. In this study, we explore inclusive perceptions that historically underrepresented groups can perform well in STEM as well as self-affiliated perceptions that your racial/ethnic group and gender group can perform well in STEM. Therefore, this study will further investigate the relationship between STEM ability perceptions and basic needs satisfaction.

### Current study

Prior studies highlight how students’ intrinsic motivation leads to persistence in STEM [22], how stereotypical perceptions can negatively influence academic intrinsic motivation [24], and how basic psychological needs, like competence, can mediate the association between stereotypical perceptions and academic outcomes such as grades [16]. However, research is needed that examines inclusive STEM ability perceptions about underrepresented or overrepresented groups and self-affiliated STEM ability perceptions about one’s social groups or other social groups. Furthermore, research has not yet examined how inclusive or self-affiliated STEM ability perceptions are related to basic psychological needs and intrinsic motivation in STEM classes. Recognizing the limitations of the previous literature, the aim of the current study is to explore the relationship between inclusive and self-affiliated perceptions of STEM abilities, basic needs satisfaction, and intrinsic motivation in STEM classes during adolescence—a critical time to maintain academic intrinsic motivation for math and science classes. Despite high intrinsic motivation being associated with positive academic outcomes [12,19,20], research has found that intrinsic motivation declines during adolescence and especially for math and science classes [10,11,25]. Therefore, by understanding what factors contribute to this decline, we can inform interventions aimed at preserving intrinsic motivation during this key time.

We also examine correlates of both gender and race/ethnicity due to the historic underrepresentation of women and ethnic minoritized groups in STEM and the higher likelihood of these groups to encounter more negative STEM perceptions [54–56]. Although there have been many studies on STEM gender beliefs, there has been less research on STEM racial/ethnic beliefs. It is important to also explore people’s perceptions of STEM abilities based on race/ethnicity, as these perceptions could be contributing to the underrepresentation of URM in STEM fields. This study utilizes two models: self-affiliated STEM ability perceptions (STEM perceptions about your racial/ethnic group and gender group vs other racial/ethnic groups and gender groups) and inclusive STEM ability perceptions (STEM perceptions about historically underrepresented groups vs overrepresented groups), to explore the role of different types of inclusive thinking about STEM.

### Hypotheses

1. As prior research has reported a positive association between basic needs satisfaction and intrinsic motivation [13], therefore in addition to the more central hypotheses below, we expect to affirm that there will be a direct positive effect between basic needs satisfaction and intrinsic motivation.
2. For the self-affiliated STEM ability perceptions model, based on previous research [24,42], we expect that having higher perceptions of your gender and racial/ethnic group’s STEM abilities will be positively associated with intrinsic motivation and basic needs satisfaction. However, having higher perceptions of other gender or racial/ethnic groups’ STEM abilities will be negatively associated with intrinsic motivation and basic needs satisfaction.
3. For the inclusive STEM ability perceptions model, based on prior literature [16] we expect that perceptions of underrepresented groups (having higher perceptions of girls and URM) will be positively associated with intrinsic motivation and basic needs satisfaction, and that perceptions of overrepresented groups (having higher perceptions of boys and White students) will be negatively associated with intrinsic motivation and basic needs satisfaction.
4. As previous research has found that basic needs satisfaction mediates the relationship between perceptions and academic outcomes [16], we expect to see a similar indirect effect of the STEM ability perceptions on intrinsic motivation through basic needs satisfaction.

Given mixed results of prior research [23,24,47], it is an open question whether racial/ethnic, gender, or age differences would emerge in our focal variables Perceptions of Your Gender Group, Perceptions of Your Racial/Ethnic Group, Perceptions of Other Gender Groups, Perceptions of Other Racial/Ethnic Groups, Perceptions of Underrepresented Groups, Perceptions of Overrepresented Groups, Basic Needs Satisfaction, and Intrinsic Motivation. Therefore, we aimed to explore differences by race/ethnicity, gender, and age.

## Methods

### Participants

Participants were high school (9^th^ – 12^th^ grade) students from five public schools in the Southeastern United States (*N* = 285, 56.1% girls, *M*_age_ = 15.76 years, Range_age_ = 13-20, *SD* = 1.24). 42% of the sample identified as Black/African American; 33% White/European-American; 10.1% Latinx; 1.6% Asian/Asian-American; 0.6% American Indian/Native American; 0.3% Native Hawaiian/Pacific Islander; 0.3% Arab/Arab-American; 9.9% Bi-racial or other; and 2.2% of the sample did not report their race/ethnicity. All five public schools received Title 1 funding, indicating they serve low-income populations. Students completed the survey between November 2020 and April 2021 and many of them were attending school primarily virtually (48.8%) due to the COVID-19 pandemic. 33.7% of participants reported attending school in a hybrid format, 17.5% reported attending school in person, and 0.4% did not report how they were attending school. Most students reported taking 1-2 STEM classes while the data for the current study was being collected. during the study.

### Measures

#### Perceptions of STEM abilities.

The measures used were adapted from prior research [8,57]. Participants were asked to drag a slider to indicate how true (0= not true at all to 100= very much true) they thought the following sentence about girls and boys were; “I think that girls/boys usually do well in STEM.” Participants were also asked to drag a slider to indicate how true (0= not true at all to 100= very much true) each sentence was about Black, Latinx, and White students; for instance, “I think that Black students usually do well in STEM.” For this measure, STEM was defined for students (i.e., “In this survey, we will use the term STEM, this refers to Science, Technology, Mathematics, and Engineering.”).

**Inclusive STEM** **ability perceptions**. As girls and URM (Black and Latinx) students are often subject to negative STEM perceptions [54–56], responses to the three ability perception items were averaged to create the perceptions about underrepresented groups measure (Perceptions of Underrepresented Groups; α =.91). The responses to the items about boys and White students were averaged to create the perceptions about overrepresented groups measure (Perceptions of Overrepresented Groups; *r* = 0.67, *p* < 0.001).

**Self-**affiliated** STEM** **ability perceptions.** The perceptions about your gender group measure (Perceptions of Your Gender Group) used girls’ responses to the perception statement about girls, and boys’ responses to the perception statement about boys. The perceptions about other gender groups measure (Perceptions of Other Gender Groups) used girls’ responses to the statement about boys, and boys’ responses to the statement about girls. The perceptions about your racial/ethnic group measure (Perceptions of Your Racial/Ethnic Group) used Black students’ responses to the statements about Black students, and the same was done for White students and Latinx students. The perceptions about other racial/ethnic groups measure (Perceptions of Other Racial/Ethnic Groups) used the average of Black students’ responses to the statements about White students and Latinx students. The same process was used for White and Latinx students’ responses.

#### Basic needs satisfaction in STEM.

This 16-item measure features three subscales that evaluated participants’ feelings of competence, autonomy, and relatedness in their STEM classes. This measure was adapted from the Basic Psychological Need Satisfaction at Work Scale used to assess need satisfaction and has been used in many studies [58–60]. Participants were given six statements about autonomy, six statements about competence, and six statements about relatedness and asked to rate how true (1 = not at all true to 7 = very true) each statement is for themselves. Examples of statements for each subscale include autonomy: “I am free to express my ideas and opinions in my STEM classes”, competence: “I have been able to learn interesting new skills in my STEM classes”, and relatedness: “I consider the people in my STEM classes to be my friends.” A latent construct capturing all 16 items was used as the Basic Needs Satisfaction variable (α =.87).

#### Intrinsic motivation.

This measure assessed participants’ intrinsic motivation (α =.88) to engage in STEM. This measure was adapted from the Situational Motivation Scale that assessed motivation for participating in an activity [61]. Participants were given four statements asking why they were engaged in STEM classes. They responded to each statement on a 7-point Likert scale (1= corresponds not at all to 7= corresponds exactly). Examples of statements were: “Because my STEM classes are fun” and “Because I think that STEM classes are interesting.”

### Procedure

This research was approved by the IRB at North Carolina State University (20526). The survey was a part of a larger study assessing students’ experiences in their STEM courses. For this study participants were asked about their STEM courses as a collection rather than individual science, technology, engineering, and mathematics courses. All students at participating schools were invited to participate and opt-out consent forms were sent home to families electronically. While 694 students who had parental consent assented to participation, we analyzed only the data from the 285 participants who completed all relevant survey measures. Participants were entered into a drawing for $10 electronic gift cards to thank them for participation.

### Data analysis

Descriptive statistics were computed first and then a correlation matrix was computed to confirm variable associations. We subsequently conducted the intraclass correlation coefficients (ICC) to determine if multilevel modeling was needed to account for students nested in schools. The ICCs for all relevant variables were between.01 and.05, indicating that multilevel modeling was unnecessary.

Next, a measurement model was computed to assess the factor structure of Basic Needs Satisfaction and Intrinsic Motivation. A path model was then estimated for self-affiliated STEM ability perceptions to analyze the association between Perceptions of Your Gender Group, Perceptions of Other Gender Groups, Perceptions of Your Racial/Ethnic Group, Perceptions of Other Racial/Ethnic Groups, and Intrinsic Motivation as well as the mediating effects of Basic Needs Satisfaction. Gender, race/ethnicity, and age were regressed onto Perceptions of Your Gender Group, Perceptions of Other Gender Groups, Perceptions of Your Racial/Ethnic Groups, Perceptions of Other Racial/Ethnic Groups, Basic Needs Satisfaction, and Intrinsic Motivation to check for differences based on demographics. A path model was also estimated for inclusive STEM ability perceptions to analyze the association between Perceptions of Underrepresented Groups, Perceptions of Overrepresented Groups, and Intrinsic Motivation as well as the mediating effects of Basic Needs Satisfaction. Gender, race/ethnicity, and age regressed onto Perceptions of Underrepresented Groups and Perceptions of Overrepresented Groups to check for differences. Analyses were conducted using M*plus* Version 8, with full information maximum likelihood estimation (FIML) [62]. Fit indices were computed and model fit was assessed following guidelines established by Hu and Bentler [63].

## Results

### Descriptive statistics

Results showed that participants had moderately high means for Perceptions of Underrepresented Groups, Overrepresented Groups, Your and Other Gender Groups, and Your and Other Racial/Ethnic Groups indicating that participants tended to believe that all students usually do well in STEM, and the means for Basic Needs Satisfaction and Intrinsic Motivation were about average (see Table 1). Additionally, all variables were correlated with each other, however Perceptions of Other Racial/Ethnic Groups was not related to Basic Needs Satisfaction (see Table 2). There were no significant gender or racial/ethnic differences among participants for any of the key variables.

**Table 1:** Descriptive statistics.

	Mean	Standard Deviation	Range
Perceptions of Underrepresented Groups	71.62	21.17	0–100
Perceptions of Overrepresented Groups	72.01	20.87	0–100
Perceptions of Your Gender Group	73.17	22.41	0–100
Perceptions of Other Gender Groups	72.29	21.85	0–100
Perceptions of Your Racial/Ethnic Group	70.31	22.58	0–100
Perceptions of Other Racial/Ethnic Groups	70.97	21.67	0–100
Basic Needs Satisfaction	4.58	0.83	2.06–6.88
Intrinsic Motivation	4.61	1.28	1–7

**Table 2:** Correlations.

Variable	1	2	3	4	5	6	7	8
1.-Perceptions of underrepresented groups	–							
2. Perceptions of overrepresented groups	0.76**	–						
3. Perceptions of your gender group	0.83**	0.81**	–					
4. Perceptions of other gender groups	0.78**	0.79**	0.59**	–				
5. Perceptions of your racial/ethnic group	0.84**	0.78**	0.82**	0.59**	–			
6. Perceptions of other racial/ethnic groups	0.90**	0.82**	0.71**	0.72**	0.72**	–		
7. Basic needs satisfaction	0.20**	0.12*	0.15*	0.19**	0.13*	0.09	–	
8. Intrinsic motivation	0.18**	0.13*	0.17**	0.18**	0.17*	0.14*	0.55**	–

*p<0.05.

**p<0.01.

### Path analysis for self-affiliated STEM ability perceptions

The perception measures (Perceptions of Your Gender Group, Perceptions of Other Gender Groups, Perceptions of Your Racial/Ethnic Group, Perceptions of Other Racial/Ethnic Groups) were regressed onto Basic Needs Satisfaction, and Basic Needs Satisfaction was regressed onto Intrinsic Motivation. Gender, race/ethnicity, and age were regressed onto the main variables. Indirect effects Perceptions of Your Gender Group, Perceptions of Other Gender Groups, Perceptions of Your Racial/Ethnic Group, Perceptions of Other Racial/Ethnic Groups to Intrinsic Motivation were also tested. Fit indices showed this model did not fit adequately: χ^2^ (295) = 963.48, p < 0.001; CFI = 0.73, TLI = 0.67; RMSEA = 0.09, [CI = 0.08, 0.10]; SRMR = 0.11. Therefore, we did not continue to explore this model. We next assessed inclusive STEM ability perceptions.

### Path analysis for inclusive STEM ability perceptions

**Fig 2:**
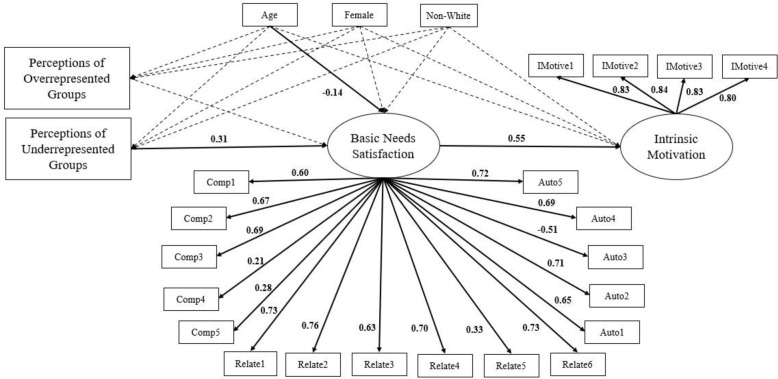
Path analysis for inclusive STEM ability perceptions.

Perceptions of Underrepresented Groups and Perceptions of Overrepresented Groups were regressed onto Basic Needs Satisfaction, and Basic Needs Satisfaction was regressed onto Intrinsic Motivation. Gender, race/ethnicity, and age were regressed onto Perceptions of Underrepresented Groups, Perceptions of Overrepresented Groups, Basic Needs Satisfaction, and Intrinsic Motivation (see Fig 2). Indirect effects of Perceptions of Underrepresented Groups to Intrinsic Motivation and from Perceptions of Overrepresented Groups to Intrinsic Motivation were also tested. Fit indices showed this model fit adequately: χ^2^ (251) = 433.70, p < 0.001; CFI = 0.92, TLI = 0.90; RMSEA = 0.05, [CI = 0.04, 0.06]; SRMR = 0.06.

Path analysis depicting the direct effects of Perceptions of Underrepresented Groups on Basic Needs Satisfaction and Basic Needs Satisfaction on Intrinsic Motivation. Regression weights for unidirectional pathways are standardized. Solid lines represent paths that were significant (*p* < 0.05). Covariances were not included in the model for clarity.

As hypothesized, there was a positive effect of Perceptions of Underrepresented Groups on Basic Needs Satisfaction, however there was no significant effect of Perceptions of Overrepresented Groups on Basic Needs Satisfaction. There was a positive effect of Basic Needs Satisfaction on Intrinsic Motivation. We also found a negative effect of age on Basic Needs Satisfactions, indicating that older students had less basic needs satisfaction than did younger students. Unexpectedly, there was not a significant direct effect of Perceptions of Underrepresented Groups or Perceptions of Overrepresented Groups on Intrinsic Motivation. However, there was a positive indirect effect of Perceptions of Underrepresented Groups on Intrinsic Motivation via Basic Needs Satisfaction (estimate = 0.17, SE= 0.004, *p* = 0.01). There was not a significant indirect effect of Perceptions of Overrepresented Groups on Intrinsic Motivation. Perceptions of Underrepresented Groups was positively associated with Intrinsic Motivation and this relationship was driven by Basic Needs Satisfaction, therefore the more students believed that girls and URM do well in STEM, the higher their basic needs satisfaction, and, consequently, the higher their intrinsic motivation.

## Discussion

This study examined the associations between self-affiliated STEM ability perceptions, inclusive STEM ability perceptions, basic needs satisfaction, and intrinsic motivation. We were not able to identify a model for self-affiliated STEM ability perceptions that fit the data adequately. However, the model for inclusive STEM ability perceptions fit well. As expected, Perceptions of Underrepresented Groups’ STEM abilities was positively related to Basic Needs Satisfaction and Basic Needs Satisfaction was positively related to Intrinsic Motivation. Additionally, there was a positive indirect effect of Perceptions of Underrepresented Groups on Intrinsic Motivation through Basic Needs Satisfaction, but unexpectedly there was not a direct effect on Intrinsic Motivation. These findings expand our knowledge by showing that inclusive thinking may play an influential role in promoting basic needs satisfaction in STEM.

### Racial, gender, and age differences

Contrary to previous findings [23,24,46,47] we did not find any gender or racial differences in any of the key variables. Our results revealed that girls and boys, and URM and White students had similar ratings of inclusive STEM ability perceptions and self-affiliated STEM ability perceptions. Therefore, adolescents are recognizing that people from different racial backgrounds and girls can do well in STEM. This is an exciting finding, as it suggests that adolescents may be increasingly aware that all students can excel in STEM domains. Past research also had mixed findings for ethnic and gender differences for intrinsic motivation [23,24] and our results showed that URM and White adolescents, as well as male and female participants in this study all had similar rates of intrinsic motivation. Our sample was quite ethnically diverse, with over 50% of participants identifying as Black or Latinx and only 33% identifying as White. Future research should consider exploring school racial composition to identify whether factors such as level of school diversity is associated with intrinsic motivation and inclusive perceptions.

Lastly, whereas past research has shown that intrinsic motivation tends to decline throughout adolescence [10,25], we did not find any age differences in intrinsic motivation, however we did find age differences in basic needs satisfaction. Older students had lower basic needs satisfaction than did younger students. This is a novel finding as past research has not yet documented age differences in students STEM needs satisfaction. This suggests that schools might attend to ensuring that students’ basic psychological needs in STEM are met as they progress through high school, taking more advanced and specialized STEM classes which may threaten their needs satisfaction. Additionally, this finding could be due to the virtual learning context that many of the students were participating in. Students may not have had as rich learning experiences online as they would have in a formal classroom. While it is unexpected that intrinsic motivation did not decline with age in our sample, this could be due to our cross-sectional design or, again, related to students’ nontraditional virtual school experiences due to COVID-19. That is, if we had tracked students’ intrinsic motivation longitudinally, we may have seen similar age differences that past research has found. Additionally, Zaccoletti, Camacho [64] documented that the pandemic may be associated with different trends in motivation. We may have found different results if the research had been conducted outside of the pandemic timeframe. Thus, future research should continue to examine age-related patterns in intrinsic motivation for STEM.

### Self-affiliated STEM ability perceptions

We first tested a model assessing the participants’ perceptions of their own and other gender and racial/ethnic groups’ STEM abilities; however, the model did not fit adequately. These results suggest that perceptions specifically related to your own social groups may not be as influential for basic needs satisfaction as inclusive STEM perceptions in general. Perhaps believing that all students, especially those who have been underrepresented in STEM (i.e., girls, Black students, and Latinx students) do well in STEM can help promote better classroom experiences, and subsequently, intrinsic motivation. Moreover, findings from a classroom-based intervention to promote inclusion amongst children, found that promoting inclusive thinking led to increases in perceptions of math and science competence beliefs for all peers [65], suggesting that it may be possible to intervene to increase positive perceptions about the abilities of students who have been underrepresented in STEM.

### The role of inclusive stem ability perceptions in self-determination

Our novel findings document the role of inclusive perceptions in shaping basic needs satisfaction. Specifically, we found that Perceptions of Underrepresented Groups positively predicted basic psychological needs. Therefore, the more students felt that girls and URM could succeed in STEM, the more they felt autonomous, competent, and related in their STEM classes. Our findings suggest that resisting traditional perceptions that depict girls and URM students as not doing well in STEM may be related to increases in students’ needs satisfaction, or vice versa. We also did not find any gender or racial/ethnic differences in any of our key variables, so having these more inclusive perceptions was beneficial for all students, not just girls and URM. Having more positive perceptions of these social groups may enable one to be more inclusive of others and feel more belonging. In fact, research has shown that girls felt more belonging in their science class when they felt that their classmates had less stereotypical beliefs [66]. Moreover, research finds that overall perceptions of inclusivity in STEM classes (for girls, boys, ethnic minority students and ethnic majority students) predicted belonging and, consequently, engagement in STEM classes for adolescents [67]. Therefore, overall recognition that groups historically excluded in STEM can do well in STEM may promote all students’ persistence in STEM and reduce some of the disparities we see in STEM. These findings suggest that future intervention work should harness the role of countering stereotypical perceptions in orienting students towards STEM success. Encouraging students to recognize that groups traditionally underrepresented in STEM can and should be successful in STEM may help to foster students’ needs satisfaction, which has been shown to be critically important for school success, including in STEM contexts [14,15,27].

Similar to previous research [13], we found a positive association between basic needs satisfaction and intrinsic motivation; when students had high feelings of competence, autonomy, and relatedness they were more likely to have higher intrinsic motivation for their STEM classes. Our findings also align with STEM specific studies that have documented the association between basic needs satisfaction and intrinsic motivation, which leads to academic benefits [14,15,21]. Outcomes such as better academic performance and lower academic stress have been associated with intrinsic motivation [12,19,20], as well as STEM persistence [15,21], which itself is especially important for girls and ethnic minoritized students who are less likely to continue with STEM subjects as they get older [5,6]. Therefore, by improving students’ needs satisfaction, we may also promote their intrinsic motivation for their STEM classes which may encourage students to take advanced STEM courses in high school, pursue STEM degrees in college, and enter the STEM workforce.

Furthermore, we did not find a direct effect of Perceptions of Underrepresented Groups on intrinsic motivation; rather, we documented a positive indirect association Perceptions of Underrepresented Groups and intrinsic motivation through students’ basic needs satisfaction. Therefore, the positive effects of inclusive thinking on intrinsic motivation can be explained through students’ basic needs satisfaction. Since needs satisfaction is an important factor for intrinsic motivation, our findings suggest that schools should implement programs focused on increasing students’ autonomy, competence, and relatedness to foster intrinsic motivation.

### Limitations

While this study provided novel findings, there are several limitations to note. First, our use of cross-sectional data limited the scope of our work, and we are not able to make causal or directional conclusions. Additionally, our data were only from a US sample, but students from other countries may also be affected by negative STEM perceptions as STEM disparities are evident globally [2]. Therefore, future research should use international samples to examine the relationship between these factors. We also only measured general needs satisfaction in STEM classes; thus, future research may benefit from analyzing students’ needs satisfaction in their science, technology, engineering, and math classes separately. These data were also collected during the COVID-19 pandemic; thus, the students were participating in school in an atypical manner (some fully virtual and some in a hybrid format) which may have impacted their needs satisfaction as well as their STEM perceptions. Further, we did not account for socioeconomic status because the sample was skewed toward low-income schools; however, this may have influenced our findings. Lastly, as our STEM ability perception measures asked explicitly about different ethnic groups and girls, participants may have answered more equitably due to social desirability effects. Future research should use both implicit and explicit measures to capture a more comprehensive representation of adolescents’ STEM ability perceptions.

## Conclusion

Building on previous research [24,36] this study shows that inclusive thinking can have positive outcomes for adolescents. It also provides new insight into key mechanisms that may be important for SDT [13,68] by showing that basic needs satisfaction can act as a mediator between STEM perceptions and intrinsic motivation. Furthermore, this study highlights that having inclusive perceptions is beneficial for all students, not only girls and URM, however, if all students have more inclusive perceptions this may allow girls and URM to feel welcomed in STEM, and thus creating more representation within these fields. Our findings suggest that schools and out-of-school programs should consider both countering stereotypic perceptions about abilities in academic domains, including STEM, and identifying strategies to increase students’ basic needs (autonomy, competence, and relatedness) to promote intrinsic motivation in STEM.

## References

[1] National Center for Science and Engineering Statistics. Women, Minorities, and Persons with Disabilities in Science and Engineering: 2021. Special Report NSF 21-321. 2021.National Center for Science and Engineering Statistics. Women, minorities, and persons with disabilities in science and engineering: 2021. Special Report; 2021.

[2] UNESCO. Girls, women and STEM: How the Ingeniosas Foundation helps discover vocations in science and technology in Chile and Latin America. 2023. Available from: https://www.unesco.org/en/articles/girls-women-and-stem-how-ingeniosas-foundation-helps-discover-vocations-science-and-technology-chile

[3] National Science Board. Science and Engineering Indicators. Arlington, VA: National Science Foundation; 2018.

[4] Patrick H, Mantzicopoulos P, Samarapungavan A, French BF. Patterns of young children’s motivation for science and teacher-child relationships. J Exp Educ 2008;76(2):121–44. doi: 10.3200/jexe.76.2.121-144

[5] Lei RF, Green ER, Leslie S-J, Rhodes M. Children lose confidence in their potential to “be scientists”, but not in their capacity to “do science”. Dev Sci. 2019;0(ja):e12837. doi: 10.1111/desc.12837 PMC6803025 31006163

[6] Wigfield A, Eccles JS, Fredricks JA, Simpkins S, Roeser RW, Schiefele U. Development of achievement motivation and engagement. In: Lamb ME, Lerner RM, editors. Handbook of Child Psychology and Developmental Science: Socioemotional Processes, Vol 3, 7th ed. Hoboken, NJ: John Wiley & Sons Inc; 2015. p. 657-700.

[7] Evans AB, Copping K, Rowley SJ, Kurtz-Costes B. Academic self-concept in black adolescents: do race and gender stereotypes matter? Self Identity 2011;10(2):263–77. doi: 10.1080/15298868.2010.485358 21552362 PMC3086770

[8] Rowley SJ, Kurtz‐Costes B, Mistry R, Feagans L. Social status as a predictor of race and gender stereotypes in late childhood and early adolescence. Social Dev 2007;16(1):150–68. doi: 10.1111/j.1467-9507.2007.00376.x

[9] Ainslie PJ, Huffman SL. Human resource development and expanding STEM career learning opportunities: exploration, internships, and externships. Adv Dev Human Resour 2018;21(1):35–48. doi: 10.1177/1523422318814487

[10] Gottfried AE, Fleming JS, Gottfried AW. Continuity of academic intrinsic motivation from childhood through late adolescence: A longitudinal study. J Educ Psychol 2001;93(1):3–13. doi: 10.1037//0022-0663.93.1.3

[11] Gottfried AE, Marcoulides GA, Gottfried AW, Oliver PH, Guerin DW. Multivariate latent change modeling of developmental decline in academic intrinsic math motivation and achievement: Childhood through adolescence. Int J Behav Dev. 2007;31(4):317–27. doi: 10.1177/0165025407077752

[12] Ryan RM, Deci EL. Self-determination Theory: Basic Psychological Needs in Motivation, Development, and Wellness. 2017.

[13] Deci EL, Ryan RM. Self-determination Theory. Handbook of Theories of Social Psychology, Vol 1. Thousand Oaks, CA: Sage Publications Ltd; 2012. p. 416–36.

[14] Brandenberger C, Hagenauer G, Hascher T. Promoting students’ self-determined motivation in Maths: results of a 1-year classroom intervention. Euro J Psychol Educ 2018;33(2):295–317.

[15] Simon R, Aulls M, Dedic H, Hubbard K, Hall N. Exploring student persistence in STEM programs: a motivational model. Canadian J Educ 2015;38(1):1–27.

[16] Plante I, de la Sablonnière R, Aronson J, Théorêt M. Gender stereotype endorsement and achievement-related outcomes: The role of competence beliefs and task values. Contemp Educ Psychol 2013;38(3):225–35.

[17] Lavigne G, Vallerand R, Miquelon P. A motivational model of persistence in science education: A self-determination theory approach. Euro J Psychol Educ 2007;22(3):351.

[18] Kiemer K, Gröschner A, Pehmer A-K, Seidel T. Effects of a classroom discourse intervention on teachers’ practice and students’ motivation to learn mathematics and science. Learn Instruct 2015;35:94–103. doi: 10.1016/j.learninstruc.2014.10.003

[19] Cerasoli CP, Nicklin JM, Ford MT. Intrinsic motivation and extrinsic incentives jointly predict performance: a 40-year meta-analysis. Psychol Bull 2014;140(4):980–1008. doi: 10.1037/a0035661 24491020

[20] Gottfried AE. Academic intrinsic motivation in elementary and junior high school students. J Educ Psychol n.d.;77(6):631–45.

[21] Froiland J, Davison M. The longitudinal influences of peers, parents, motivation, and mathematics course-taking on high school math achievement. Learn Indiv Diff 2016;50252–9.

[22] Froiland J, Worrell FC. Intrinsic motivation, learning goals, engagement, and achievement in a diverse high school. Psychol Schools 2016;53(3):321–36.

[23] D’Lima GM, Winsler A, Kitsantas A. Ethnic and gender differences in first-year college students’ goal orientation, self-efficacy, and extrinsic and intrinsic motivation. J Educ Res 2014;107(5):341–56. doi: 10.1080/00220671.2013.823366

[24] Gillen-O’Neel C, Ruble DN, Fuligni AJ. Ethnic stigma, academic anxiety, and intrinsic motivation in middle childhood. Child Dev. 2011;82(5):1470–85. doi: 10.1111/j.1467-8624.2011.01621.x 21883152 PMC3170141

[25] Otis N, Grouzet F, Pelletier L. Latent motivational change in an academic setting: A 3-year longitudinal study. J Educ Psychol 2005;97(2):170–83.

[26] Harter SA. new self-report scale of intrinsic versus extrinsic orientation in the classroom: Motivational and informational components. Dev Psychol. 1981;17(3):300–12. doi: 10.1037/0012-1649.17.3.300

[27] Gnambs T, Hanfstingl B. The decline of academic motivation during adolescence: an accelerated longitudinal cohort analysis on the effect of psychological need satisfaction. Educ Psychol 2015;36(9):1691–705. doi: 10.1080/01443410.2015.1113236

[28] Moore ME, Vega DM, Wiens KM, Caporale N. Connecting theory to practice: Using self-determination theory to better understand inclusion in STEM. J Microbiol Biol Educ. 2020;21(1):21–1.32. doi: 10.1128/jmbe.v21i1.1955 32431768 PMC7195163

[29] Correll SJ. Constraints into Preferences: Gender, Status, and Emerging Career Aspirations. Am Sociol Rev. 2004;696(1):93–113. doi: 10.1177/000312240406900106

[30] Nosek BA, Smyth FL, Sriram N, Lindner NM, Devos T, Ayala A, et al. National differences in gender-science stereotypes predict national sex differences in science and math achievement. Proc Natl Acad Sci U S A. 2009;106(26):1059–7. doi: 10.1073/pnas.0809921106 19549876 PMC2705538

[31] Jaxon J, Lei RF, Shachnai R, Chestnut EK, Cimpian A. The acquisition of gender stereotypes about intellectual ability: intersections with Race. J Soc Issues 2019;75(4):1192–215. doi: 10.1111/josi.12352

[32] Bian L, Leslie S-J, Cimpian A. Gender stereotypes about intellectual ability emerge early and influence children’s interests. Science. 2017;355(6323):389–91. doi: 10.1126/science.aah6524 28126816

[33] Starr C, Simpkins S. High school students’ math and science gender stereotypes: relations with their STEM outcomes and socializers’ stereotypes. Soc Psychol Educ 2021;24(1):273–98.

[34] Master A. Gender stereotypes influence children’s STEM motivation. Child Dev Persp. 2021;15(3):203–10.

[35] Steele CMA. Threat in the air. How stereotypes shape intellectual identity and performance. Am Psychol. 1997;52(6):613–29. doi: 10.1037//0003-066x.52.6.613 9174398

[36] Shapiro J, Williams A. The role of stereotype threats in undermining girls’ and women’s performance and interest in STEM fields. Sex Roles 2012;66(3–4):175–83.

[37] Spencer SJ, Logel C, Davies PG. Stereotype Threat. Annu Rev Psychol. 2016;67(1):415–37. doi: 10.1146/annurev-psych-073115-103235 26361054

[38] Galdi S, Cadinu M, Tomasetto C. The roots of stereotype threat: When automatic associations disrupt girls’ math performance. Child Dev 2014;85(1):250–63. 23713580 10.1111/cdev.12128

[39] Schmader T. Gender identification moderates stereotype threat effects on women’s math performance. J Exp Soc Psychol 2002;38(2):194–201. doi: 10.1006/jesp.2001.1500

[40] Gonzales P, Blanton H, Williams K. The effects of stereotype threat and double-minority status on the test performance of Latino women. Pers Soc Psychol Bull. 2002;28(5):659–70.

[41] Nadler J, Clark M. Stereotype threat: A meta-analysis comparing African Americans to Hispanic Americans. J App Soc Psychol 2011;41(4):872–90.

[42] Good C, Rattan A, Dweck CS. Why do women opt out? Sense of belonging and women’s representation in mathematics. J Pers Soc Psychol. 2012;102(4):700–17. doi: 10.1037/a0026659 22288527

[43] Delisle M-N, Guay F, Senécal C, Larose S. Predicting stereotype endorsement and academic motivation in women in science programs: a longitudinal model. Learn Indiv Diff 2009;19(4):468–75.

[44] Nehmeh G, Kelly AM. Facilitating the self-determination of undergraduate women in physics: the role of external validation. Res Sci Technol Educ 2020;39(3):306–27. doi: 10.1080/02635143.2020.1740668

[45] Copping KE, Kurtz-Costes B, Rowley SJ, Wood D. Age and race differences in racial stereotype awareness and endorsement. J Appl Soc Psychol. 2013;43(5):971–80. doi: 10.1111/jasp.12061 23729837 PMC3667990

[46] Burnett M, Kurtz-Costes B, Vuletich HA, Rowley SJ. The development of academic and nonacademic race stereotypes in African American adolescents. Dev Psychol. 2020;56(9):1750–9. doi: 10.1037/dev0001071 32658501

[47] McKown C, Weinstein RS. The development and consequences of stereotype consciousness in middle childhood. Child Dev. 2003;74(2):498–515. doi: 10.1111/1467-8624.7402012 12705569

[48] Bartini M. Gender role flexibility in early adolescence: developmental change in attitudes, self-perceptions, and behaviors. Sex Roles 2006;55(3-4):233–45. doi: 10.1007/s11199-006-9076-1

[49] Alfieri T, Ruble DN, Higgins ET. Gender stereotypes during adolescence: developmental changes and the transition to junior high school. Dev Psychol. 1996;32(6):1129–37. doi: 10.1037//0012-1649.32.6.1129

[50] Way N, Hernández MG, Rogers LO, Hughes DL. “I’m Not Going to Become No Rapper”. J Adolesc Res 2013;28(4):407–30. doi: 10.1177/0743558413480836

[51] Santos CE, Galligan K, Pahlke E, Fabes RA. Gender-typed behaviors, achievement, and adjustment among racially and ethnically diverse boys during early adolescence. Am J Orthopsychiatry 2013;83(2 Pt 3):252–64. doi: 10.1111/ajop.12036 23889017

[52] Selimbegovic L, Chatard A, Mugny G. Can we encourage girls’ mobility towards science-related careers? Disconfirming stereotype belief through expert influence. Eur J Psychol Educ. 2007;22(3):275–90. doi: 10.1007/bf03173426

[53] van Breen J, Spears R, Kuppens T, de Lemus S. Subliminal gender stereotypes: Who can resist? Pers Soc Psychol Bull. 2018;44(12):1648–63. 29781373 10.1177/0146167218771895

[54] Gaertner SL, McLaughlin JP. Racial Stereotypes: Associations and Ascriptions of Positive and Negative Characteristics. Soc Psychol Quar 1983;46(1):23. doi: 10.2307/3033657

[55] Kurtz-Costes B, Copping KE, Rowley SJ, Kinlaw CR. Gender and age differences in awareness and endorsement of gender stereotypes about academic abilities. Euro J Psychol Educ 2014;29(4):603–18.

[56] Chen X. STEM Attrition: College Students’ Paths Into and Out of STEM Fields. National Center for Education Statistics; 2013.

[57] Liben LS, Bigler RS. The developmental course of gender differentiation: Conceptualizing, measuring and evaluating constructs and pathways. Monographs of the Society for Research in Child Development. Monogr Soc Res Child Dev. 2002;67(2):i–viii, 1–47; discussion 148-83. 12465575

[58] Deci EL, Ryan RM, Gagné M, Leone DR, Usunov J, Kornazheva BP. Need satisfaction, motivation, and well-being in the work organizations of a former Eastern bloc country: A cross-cultural study of self-determination. Pers Soc Psychol Bull. 2001;27(8):930–42. doi: 10.1177/0146167201278002

[59] Ilardi BC, Leone D, Kasser T, Ryan RM. Employee and Supervisor Ratings of Motivation: Main Effects and Discrepancies Associated with Job Satisfaction and Adjustment in a Factory Setting1. J Appl Soc Psychol 1993;23(21):1789–805. doi: 10.1111/j.1559-1816.1993.tb01066.x

[60] Kasser T, Davey J, Ryan RM. Motivation and employee-supervisor discrepancies in a psychiatric vocational rehabilitation setting. Rehab Psychol 1992;37(3):175–88. doi: 10.1037/0090-5550.37.3.175

[61] Guay F, Vallerand RJ, Blanchard C. Motiv Emot 2000;24(3):175–213. doi: 10.1023/a:1005614228250

[62] Muthén LK, Muthén BO. Mplus User’s Guide. 7th Edition. Los Angeles, CA: Muthén & Muthén; 2013.

[63] Hu L-T, Bentler PM. Cut-off criteria for fit indexes in covariance structure analysis: Conventional criteria versus new alternatives. Struct Equ Model. 1999;6(1):1–55. doi: 10.1080/10705519909540118

[64] Zaccoletti S, Camacho A, Correia N, Aguiar C, Mason L, Alves RA, et al. Parents’ perceptions of student academic motivation during the covid-19 lockdown: a cross-country comparison. Front Psychol. 2020;11592670. doi: 10.3389/fpsyg.2020.592670 33391114 PMC7775314

[65] Killen M, Burkholder A, D’Esterre A, Sims R, Glidden J, Yee K. Testing the effectiveness of the Developing Inclusive Youth program: a multi-site randomized control trial. Child Dev. 2022. 10.1111/cdev.13785 PMC9179087 35612354

[66] Barth JM, Masters SL, Parker JG. Gender stereotypes and belonging across high school girls’ social groups: beyond the STEM classroom. Soc Psychol Educ 2022;25(1):275–92.

[67] Mulvey KL, Mathews C J, Knox J, Joy A, Cerda-Smith J. The role of inclusion, discrimination, and belonging for adolescent Science, Technology, Engineering and Math engagement in and out of school. J Res Sci Teach. 2022;59(8):1447–64.

[68] Deci EL, Ryan RM. Intrinsic motivation and self-determination in human behavior. New York: Plenum; 1985.

